# Exposure to Ambient Fine Particulate Air Pollution in Utero as a Risk Factor for Child Stunting in Bangladesh

**DOI:** 10.3390/ijerph15010022

**Published:** 2017-12-23

**Authors:** Nihit Goyal, David Canning

**Affiliations:** 1 Lee Kuan Yew School of Public Policy, National University of Singapore, Singapore 259772, Singapore; dcanning@hsph.harvard.edu; 2 Department of Global Health and Population, Harvard T.H. Chan School of Public Health, Boston, MA 02115, USA

**Keywords:** ambient air pollution, Bangladesh Demographic and Health Survey (BDHS), child anthropometric failure, fine particulate matter (PM_2.5_), Geographic Information System (GIS), small birth size, stunting, underweight, wasting

## Abstract

Pregnant mothers in Bangladesh are exposed to very high and worsening levels of ambient air pollution. Maternal exposure to fine particulate matter has been associated with low birth weight at much lower levels of exposure, leading us to suspect the potentially large effects of air pollution on stunting in children in Bangladesh. We estimate the relationship between exposure to air pollution in utero and child stunting by pooling outcome data from four waves of the nationally representative Bangladesh Demographic and Health Survey conducted between 2004 and 2014, and calculating children’s exposure to ambient fine particulate matter in utero using high resolution satellite data. We find significant increases in the relative risk of child stunting, wasting, and underweight with higher levels of in utero exposure to air pollution, after controlling for other factors that have been found to contribute to child anthropometric failure. We estimate the relative risk of stunting in the second, third, and fourth quartiles of exposure as 1.074 (95% confidence interval: 1.014–1.138), 1.150 (95% confidence interval: 1.069–1.237, and 1.132 (95% confidence interval: 1.031–1.243), respectively. Over half of all children in Bangladesh in our sample were exposed to an annual ambient fine particulate matter level in excess of 46 µg/m^3^; these children had a relative risk of stunting over 1.13 times that of children in the lowest quartile of exposure. Reducing air pollution in Bangladesh could significantly contribute to the Sustainable Development Goal of reducing child stunting.

## 1. Introduction

Stunting has been associated with long term physical and cognitive deficits in children, and health and economic disadvantages in later life [1]. In 2012, the World Health Assembly set a target of a 16% prevalence rate for stunting by 2025, a 40% reduction in the number of children stunted. This target has since been adopted as a Sustainable Development Goal [2]. Reaching this goal will require investment in child nutrition and prevention of infectious disease [3]. We argue, however, that outdoor (or ambient) air pollution is an additional important risk factor for stunting, and that reducing exposure to air pollution could have a significant impact on the prevalence of stunting.

Indoor air pollution from the burning of biomass for cooking has already been recognized as an important risk factor for child stunting [4]. Ambient air pollution has been rising in developing countries, and this can have a significant impact on human health [5]. In particular, it can affect the health of children [6,7] through inhalation, but also, more importantly, transplacental transmission in utero. There is substantial evidence of large effects of ambient air pollution on the birth weight of children, both in developed and developing countries [8,9,10]. Low birth weight is, in turn, linked to stunting in children; not all of the initial deficit is made up after birth [4,11,12]. We therefore suspect that ambient air pollution may be an important factor in explaining child stunting in developing countries. 

Though Bangladesh has seen an improvement in child stunting—the prevalence of stunting in children under age 5 has reduced from about 70% in the 1980s to about 40% in 2014 [13]—it still remains well above the Sustainable Development Goal target of 16%. We focus on Bangladesh because it ranked last globally on air quality in the 2016 Environmental Performance Index [14]. Among various pollutants, particulate matter of median aerodynamic diameter of 2.5 µm or less (fine particulate matter, or PM_2.5_) is thought to have the most consistent and harmful effect on health [15]. The geometric mean annual PM_2.5_ concentration in Bangladesh increased from 33.8 µg/m^3^ in 1998 to 53.5 µg/m^3^ in 2014, exposing a population of about 160 million to ambient PM_2.5_ concentrations far exceeding the World Health Organization (WHO) guideline of 10 µg/m^3^ [16]. The primary sources of ambient air pollution in Bangladesh include industries, especially brick factories, and motorized road transport [17,18]. 

Although household air pollution is a more immediate threat due to the harm from burning of solid (biomass) cooking fuel in developing countries [19], ambient air pollution is likely to become a more severe risk in the future because of increasing urban air pollution [20].

We focus on the effect of ambient air pollution on child stunting, though air pollution has much wider health effects [21]. As children are highly susceptible to air pollution [22], air pollution has been linked to child mortality and respiratory infections as well [6,23]. A systematic review of 74 studies found an effect of ambient PM_2.5_ on acute lower respiratory infection (ALRI), though most studies were conducted in North America and Europe, which have much lower levels of exposure [15]. Estimation of the global burden imposed by air pollution on health is usually modeled using concentration-response curves (CRCs) that are extrapolated from studies conducted in settings with low levels of pollution [24]. Goldizen, Sly and Knibbs [6] have emphasized the need for further research on the effects on child heath in regions with higher levels of pollution.

The objective of this study is to directly estimate the relationship between exposure to ambient PM_2.5_ in utero and child stunting in Bangladesh. The study makes several contributions to the field. First, it provides evidence on the effect on child health over a higher range of ambient annual PM_2.5_ concentration levels (20–73 µg/m^3^) than seen in many existing studies. Second, it estimates the effect of air pollution on child stunting, rather than birth weight. This is important for understanding how controlling air pollution can contribute to the sustainable development goal of reducing child stunting.

## 2. Materials and Methods

**Figure 1:**
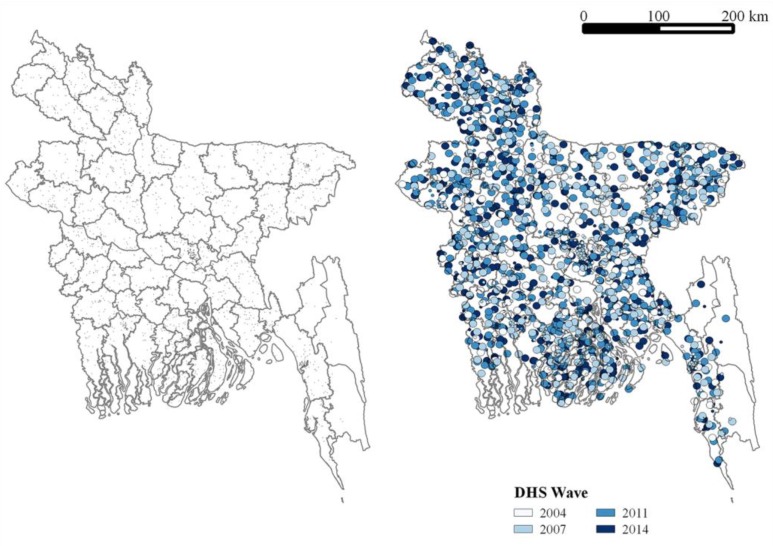
The point location (left) and buffer zone (right) of each Demographic and Health Survey (DHS) cluster on a district map of Bangladesh. The radius of the buffer zone is 5 km for a rural cluster and 2 km for an urban cluster. The color of the buffer zone indicates the DHS wave during which it was surveyed.

The study was conducted in accordance with the Declaration of Helsinki, and the protocol was approved by the Institutional Review Board at the National University of Singapore (A-16-161). Measures on cross-sections of children under age five were obtained from the Bangladesh Demographic and Health Surveys (DHS) conducted in 2004, 2007, 2011, and 2014. The DHS are nationally representative household surveys that measure indicators on marriage, fertility, family planning, reproductive health, child health, and human immunodeficiency virus infection and acquired immune deficiency syndrome (HIV/AIDS) [25]. The DHS randomly sample clusters of households, with clusters being selected from census enumeration areas, stratified by region and rural and urban areas, and then interview women of reproductive age in about 30 households per cluster [26]. The GPS coordinates of each cluster are recorded and reported, though with some noise to protect household privacy [27]. The data are available upon registration and request from the DHS Program. Figure 1 shows the distribution of clusters by DHS wave in our sample.

Comprehensive direct measures of ambient PM_2.5_ concentration based on ground-level monitoring are not available for Bangladesh. As an alternative, we use estimates of the ambient annual average PM_2.5_ concentration at a resolution of 0.01° × 0.01° (approximately, a 1 km × 1 km grid) over the period 1998–2014 calculated from satellite information, which has been calibrated to match available ground-based monitoring measures [16]. Earlier versions of the estimates of ambient PM_2.5_ concentration based on satellite data were found to be highly correlated with, but systematically lower than, the results from ground based monitoring in the Indian Subcontinent, particularly at high concentration levels [28]. However, the more recent version of the data we use has already been bias corrected to match available ground based monitoring; these bias-adjusted estimates have a correlation of 0.81 with the ground based monitoring data. Our approach is similar to that used to estimate air pollution as a risk factor for the Global Burden of Diseases (GBD) study [16,29].

We focus on four outcomes: stunting, wasting, underweight, and birth size. Stunting is defined as a height-for-age z-score (HAZ) of less than −2. The HAZ is calculated by taking the child’s height, minus the median height of well-nourished children of the same age in a reference population (defined by the WHO), and dividing this difference by the standard deviation of the age specific reference group. Similarly, underweight is defined as a weight-for-age z-score (WAZ) of less than −2, while wasting is defined as a weight-for-height z-score (WHZ) of less than −2. The methodology used and data quality of these anthropometric measures have been studied [30]. Birth size, as reported by the mother, is recorded on a five-point scale in the DHS, varying from very large to very small. For this study, small birth size is defined as the lower two categories, i.e., smaller than average or very small. Unfortunately, the Bangladesh DHS does not collect data on birth weight, which would be a more reliable, objective measure. The subjective reporting of birth size on a five-point scale may contain large measurement error. 

The summary statistics for outcomes are presented in Table 1. We see a decline in stunting and underweight over time though wasting persists at a fairly constant level. The proportion of children with small birth size seems to hold fairly steady, though we only have data for the latter two surveys.

The main explanatory variable for this study is the ambient PM_2.5_ level in microgram per cubic meter (µg/m^3^) that the child is exposed to in utero. Although there is debate on the specific timing of exposure most relevant to child morbidity and mortality, the effects of prenatal and neonatal exposure have been found to be most important and lasting due to high sensitivity during fetal development and early life [6,31]. The PM_2.5_ exposure we measure at different locations and times may be composed of particles from different sources, with somewhat different sizes, and different chemical compositions. Such compositional factors may affect the toxicity and health effects of the particulate matter [32]. One possible biological mechanism is thought to be through Polycyclic aromatic hydrocarbons (PAH) in the particulate matter entering the mother’s bloodstream, where it can lead to slow fetal development and low birthweight [33], which we hypothesize then leads to child stunting. Moreover, high sulfur content in PM_2.5_ concentrations, holding constant the overall concentration, has been recently found to be associated with low birth weight in children [34]. Unfortunately, we do not have measures of the source, or the composition, of particulate matter in our measurement of ambient PM_2.5_ concentration level. Thus, while we find an effect of PM_2.5_ exposure in utero on child stunting overall, we are likely to be estimating an average effect, with some heterogeneity of impact being related to particulate composition. Measures of exposure to air pollution after birth were not found to significantly worsen child health outcomes in our data.

Our estimates of the exposure to ambient PM_2.5_ in utero for each child in our sample were obtained by matching the location information in the Bangladesh DHS with the geographically specific annual average PM_2.5_ concentration, without dust and sea-salt [35], in the period the child was in utero. Matching was carried out using QGIS software (version 2.12.3-Lyon; Open Source Geospatial Foundation Project, Beaverton, OR, USA). The noise added to the DHS location data to protect respondent confidentiality may introduce a bias in spatial analysis of DHS data [36]. Most urban clusters are displaced by up to 2 km, while rural clusters are displaced by up to 5 km with a distance selected from a uniform distribution. We therefore constructed a buffer zone of 2 km around each reported urban location and 5 km around each reported rural location and calculated the average PM_2.5_ level in this buffer zone. In practice, the PM_2.5_ concentration at the reported cluster location and the concentration averaged over the buffer zone are highly correlated (*ρ* > 99.9%); the noise added to the location data is unlikely to bias our analysis. For our analysis, however, we use the average PM_2.5_ concentration level, while the child is in utero, in the buffer zone around the reported cluster location. Note that since children in each wave of the DHS survey can be aged up to five years old, we require exposure data from 1998 to 2014 to measure exposure in utero.

Various functional forms for the effect of ambient PM_2.5_ level on health outcomes have been proposed. A linear relationship below 30 µg/m^3^ or 50 µg/m^3^ of PM_2.5_ and flat thereafter (Lin30 and Lin50, respectively) has been suggested [37,38], and a logarithmic relationship has also been put forward [15]. Burnett, et al. [39] have proposed a highly non-linear dose response function covering ambient air pollution, indoor air pollution, and tobacco smoke, though this has a much wider range of exposures than seen in our study. Rather than impose a functional form, we report results for a model where we divide exposure into four quartiles and estimate the effect of exposure in each quartile. Each quartile accounts for the exposure level of 25% of the children in our sample. 

**Figure 2:**
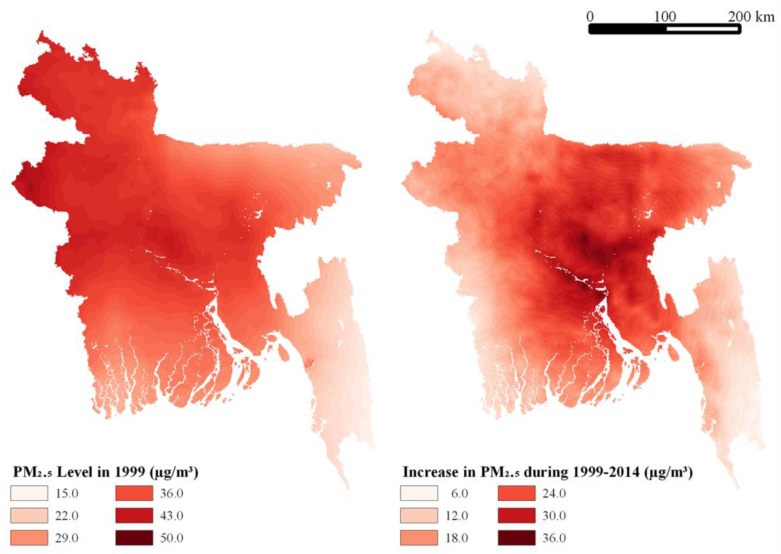
PM_2.5_ level (µg/m^3^) in 1999 (left) and increase in PM_2.5_ level (µg/m^3^) between 1999 and 2014 (right) in Bangladesh.

The mean in utero PM_2.5_ level for the study sample was 45.7 µg/m^3^, with the means of the four waves rising steadily from 42.0 in 2004 to 48.44 in 2014. The PM_2.5_ level in utero was computed by calculating the PM_2.5_ level in the nine months before birth, taking a weighted average of annual PM_2.5_ level when the in utero period spanned two calendar years. The spatial distribution of ambient PM_2.5_ level in Bangladesh in 1999 and an increase in ambient PM_2.5_ level between 1999 and 2014 is shown in Figure 2. The in utero PM_2.5_ level varied from a minimum of 19.6 µg/m^3^ in 1999 for a child in the Cox’s Bazar district of Chittagong division to a maximum of 72.9 µg/m^3^ in 2014 for a child in Dhaka district of Dhaka division. Our four quartiles of exposure in the same are less than 40.3 µg/m^3^, the interval 40.3–45.8 µg/m^3^, the interval 45.8–51.9 µg/m^3^, and over 51.9 µg/m^3^ (Table 1). Our results suggest rising risk of anthropometric failure with exposure over the first three quartiles but similar risks for exposure in the third and fourth quartiles, which is consistent with the Lin50 model. 

We use characteristics that have been identified as risk factors for child stunting in recent studies as covariates [4,40]. Risk factors can be classified into different groups as child, parental, household, and environmental factors. Child level controls in our analysis include the age of the child, indicators for the birth interval from the previous birth, which is an indicator for a twin or other multiple birth, the sex of the child, an indicator for birth order (whether first born), and a report of diarrhea in the two weeks before the survey. Parental characteristics include short maternal stature (height less than 160 cm), maternal underweight (body mass index, or BMI, less than 18.5 kg/m^2^), indicators for teenage motherhood, and the mother’s and partner’s education levels at the time of survey. Household characteristics include the type of residence of the household (whether urban) and a measure of the wealth quintile of the household based on an asset index. Environmental characteristics are indicators for the presence of an improved water source, presence of improved sanitation, and type of cooking fuel in household. The use of solid fuel biomass for cooking, and resultant indoor air pollution has been identified as an important risk factor for health [4]. In addition to household level variables, we add fixed effects for the 64 districts of Bangladesh and survey year fixed effects as covariates to account for unobserved district level effects [41] and trends in child anthropometric outcomes over time. Note that the use of survey year fixed effects, and our inclusion of the child’s age at the time of the survey, means that a time trend in the child’s birth date would be collinear with our existing model. Data on the location of district boundaries were obtained from the GADM spatial database on the world’s administrative areas [42], and were combined with DHS data using QGIS software. 

The Bangladesh DHS has 29,697 observations on children under 5 (6908 in 2004, 6150 in 2007, 8753 in 2011, and 7886 in 2014) from 1922 clusters (361 each in 2004 and 2007, and 600 each in 2011 and 2014). The study sample was smaller due to missing information. First, observations that lacked geocoded data (*n* = 42) were dropped. Subsequently, observations with missing information on one or more of short birth interval (*n* = 72), maternal short stature (*n* = 395), maternal underweight (*n* = 403), education level of the mother (*n* = 3), education level of the mother’s partner (*n* = 20), improved water in the household (*n* = 2155), improved sanitation in the household (*n* = 2155), type of cooking fuel in household (*n* = 2748), and district (*n* = 177) were excluded from the regression. Additionally, observations with missing information on childhood diarrhea (*n* = 1684) were excluded from the regression of stunting, wasting, and underweight (but not small birth size). Finally, observations with missing information on stunting (*n* = 3138), wasting (3137), underweight (3138), and small birth size (*n* = 14,466) were excluded for the respective regression. This resulted in a final sample of 23,187 children for regression of stunting, 23,188 children for regression of wasting, 23,187 children for regression of underweight, and 11,870 children for regression of small birth size.

Following Zou [43], we estimate the effect of ambient air pollution on our outcome variables using modified Poisson regression with robust standard errors. The coefficients can be interpreted as adjusted relative risk ratios. Standard errors are clustered at the DHS cluster level to account for the cluster randomized sampling methodology. This allows for correlation between the outcomes for children in the same cluster. The analyses were run using STATA software (version STATA/SE 14.1; StataCorp LP, College Station, TX, USA). Given the presence of district fixed effects and survey year fixed effects, the identification of the effect of air pollution on our outcomes depends on the level of exposure of a child relative to the average level of air pollution in their district at the time. Specifications with random effects, that allow for comparison of pollution levels across districts are given in the supplementary appendix, though these may be biased if there are other unobserved factors at the district level that affect child health. 

## 3. Results

The results of the regression are presented in Table 2. The reference group for the analysis is children with exposure in the lowest quartile, with ambient concentrations of PM_2.5_ less than 40.3 µg/m^3^ while in utero. The results in column 2 of Table 2 indicate that children exposed to higher levels of ambient air pollution are more likely to be stunted. Children in the second quartile of exposure, at levels in the range 40.3–45.8 µg/m^3^ have a relative risk of stunting of 1.074 (95% confidence interval: 1.014–1.138). The third quartile of exposure has a higher relative risk of stunting of 1.150 (95% confidence interval: 1.069–1.237), and the fourth quartile has a similarly high risk of stunting. The leveling-off of the effect between the third and fourth quartiles, which divide at a level of 52.0 µg/m^3^, is consistent with the Lin50 model, which has linear effects up to 50.0 µg/m^3^ and is flat thereafter. 

The results for our covariates are in line with previous studies. The risk of stunting is lower for children with highly educated parents and in households in higher wealth quintiles. Stunting is more likely if the mother is very young, and has low stature or BMI. First births, a short birth interval, and diarrhea are also associated with an increased risk of stunting. Cooking with biomass or solid fuel, which is linking to indoor air pollution, has a somewhat elevated the risk of stunting, but the effect is not statistically significant. This finding is similar to the findings on the effect of cooking with solid fuel on child mortality in previous studies on Bangladesh [44]. 

**Figure 3:**
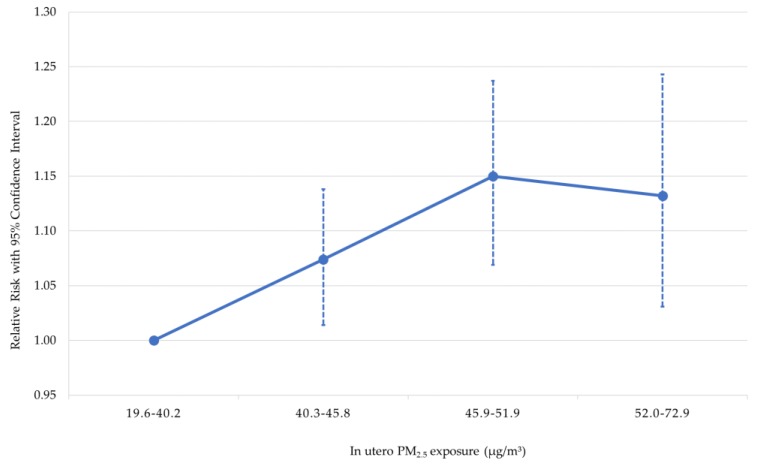
Adjusted relative risk for stunting by quartile of in utero PM_2.5_ exposure.

Figure 3 shows the adjusted relative risk of stunting by quartile of in utero PM_2.5_ exposure. The results for wasting and underweight are similar to those that are found for stunting, with significantly higher relative risk of poor anthropometric outcome with increasing in utero exposure to ambient air pollution, but with some evidence of a leveling-off of the effect between the third and fourth quartiles. For small birth size, we find higher point estimates of relative risk as the pollution level increases, but the 95% confidence intervals always include 1, and, thus, it is unclear whether the results are different from no effect. However, this may be due to subjectivity and measurement error produced in the variable due to self-reporting.

## 4. Discussion

The results indicate that children exposed to a higher level of ambient PM_2.5_ in utero are at higher risk of anthropometric failure, even after accounting for various confounding characteristics. The findings are particularly important given the increase in PM_2.5_ levels since 1999. The increase in mean PM_2.5_ level in birth year in the sample (from 34.50 µg/m^3^ in 1999 to 51.06 µg/m^3^ in 2014) is equivalent to a shift from the lowest quartile of exposure to the third quartile, and can be associated with an increase in relative risk of stunting by 1.150. Our results imply that if ambient fine particulate pollution had been in the lowest quartile (19.6–40.2 µg/m^3^) for all of Bangladesh, then the stunting prevalence in 2014 would have been 27.3% instead of 30%. Decreasing ambient PM_2.5_ levels to the WHO guideline of 10 µg/m^3^ might bring even larger gains in terms of reduced child stunting; however, the lowest recorded exposure level in our sample is 19.6 µg/m^3^ and the effects of lowering exposure levels to below the WHO guideline would require extrapolation of our results out of sample. Nevertheless, our findings suggest that rising levels of ambient air pollution in Bangladesh have slowed the improvement in child development and that reducing these high levels of pollution could contribute to better child health. 

Our results complement studies that find an effect of indoor air pollution due to cooking with biomass. Mishra and Retherford [45] have found that the prevalence of severe stunting was higher among children from households using biofuel for cooking. A more recent study by Kyu, et al. [46], using a seven country DHS data between 2005 and 2007, has estimated that biofuel smoke, of which PM_2.5_ is a major constituent, from household cooking was associated with stunting. 

Our study has several limitations. Exposure to other forms of ambient air pollution may be important [47]. Jedrychowski, et al. [48] found an effect of variation in sulphur dioxide (SO_2_) and suspended particulate matter (SPM) on child height in Krakow, Poland. Bobak, et al. [49] observed that the height of children was negatively associated with air pollution from coal burning in Britain in 1946. However, we lack satisfactory data on these other forms of pollution in Bangladesh. The exposure of this study, ambient PM_2.5_ level may have been subject to measurement error due to the limited ground-based monitoring of air pollution in South Asia for calibration of the satellite data to ground level exposure [16]. In addition, diurnal and seasonal variability in PM_2.5_ level in utero might be relevant for child health; however, information on diurnal or seasonal variability of ambient PM_2.5_ was not available in the data used for this study.

Although we control for the use of biomass fuel for cooking—typically, the main source of household air pollution in developing countries—data on the actual PM_2.5_ level inside the residence from household monitors would have provided a more accurate estimate [50]. Also, our data suffers from missing observations: around 20% of our observations are lost due to missing outcome or covariate data for regression on child stunting, wasting, and underweight. We assume that these observations are missing at random; if not, there may be a selection bias in our estimates. Child size at birth was not asked in the first two survey waves, which gives us a much smaller sample size for this regression. In any case, direct measures of child weight at birth would be preferable to these subjective self-reported data by mothers. In addition, we match children to exposure in utero based on their residence at the time of the survey, which will mean that our exposure measures are inappropriate for children who moved in from other areas.

Limitations notwithstanding, with its choice of DHS data for child health and high spatial resolution PM_2.5_ data for ambient air pollution, this study was based on a pooled cross-section of four nationally representative waves, over a long time-period, and a wide range of ambient PM_2.5_ concentration levels. Further, it controlled for common child, parental, household, and environmental characteristics, and it incorporated district dummies and survey year fixed effects to account for spatial and temporal variations in unobserved factors.

## 5. Conclusions

The purpose of this study was to estimate the relationship between ambient PM_2.5_ and child stunting in Bangladesh. The results showed that exposure to a high level of PM_2.5_ in utero is associated with an increase in the relative risk of stunting, wasting, and underweight. In our sample, in 2014, 30% of the children under age 5 in Bangladesh were stunted. Given that over 10 million children were born in Bangladesh over the last five years [51], our estimates imply that reducing ambient air pollution in all of Bangladesh to levels seen in the lowest quartile of exposure would have reduced the number of children who were stunted by as much as 270,000.

## Figures and Tables

**Table 1:** Summary statistics for prevalence of outcomes and distribution of exposure by the Bangladesh Demographic and Health Survey (BDHS) wave.

Variable	*N*	BDHS 2004	BDHS 2007	BDHS 2011	BDHS 2014	Total
*Outcome*						
Stunting	23,187	0.43	0.36	0.35	0.30	0.36
Wasting	23,188	0.13	0.16	0.15	0.13	0.14
Underweight	23,187	0.47	0.46	0.43	0.40	0.44
Small birth size	11,870	-	-	0.18	0.19	0.18
*In utero PM_2.5_ (µg/m^3^)*						
19.6–40.2 µg/m^3^	6617	0.40	0.25	0.21	0.17	0.25
40.3–45.8 µg/m^3^	6593	0.26	0.27	0.26	0.21	0.25
45.9–51.9 µg/m^3^	6512	0.22	0.26	0.26	0.26	0.25
52.0–72.9 µg/m^3^	6614	0.12	0.22	0.27	0.37	0.25

Values are proportion of children in the dataset. Information on birth size is not available in BDHS 2004 and BDHS 2007.

**Table 2:** Association between in utero PM_2.5_ exposure and child anthropometric failure.

Variable, Outcome	Stunting	Wasting	Underweight	Small Birth Size
PM_2.5_ exposure in utero, 40.3–45.8 µg/m^3^	1.074 [1.014, 1.138]	1.109 [0.990, 1.243]	1.118 [1.063,1.176]	1.040 [0.879, 1.230]
PM_2.5_ exposure in utero, 45.9–51.9 µg/m^3^	1.150 [1.069, 1.237]	1.247 [1.082, 1.437]	1.156 [1.087, 1.231]	1.100 [0.906, 1.335]
PM_2.5_ exposure in utero, 52.0–72.9 µg/m^3^	1.132 [1.031, 1.243]	1.272 [1.069, 1.512]	1.127 [1.041, 1.220]	1.209 [0.965, 1.516]
Age of child (months)	1.013 [1.012, 1.014]	1.002 [1.000, 1.004]	1.011 [1.010, 1.012]	-
Twin or triplet	1.615 [1.424, 1.831]	0.957 [0.659, 1.390]	1.500 [1.339, 1.679]	2.205 [1.720, 2.827]
Female	1.048 [1.014, 1.083]	1.017 [0.955, 1.083]	1.092 [1.062, 1.123]	1.188 [1.105, 1.278]
First child	0.968 [0.918, 1.021]	0.895 [0.812, 0.986]	0.966 [0.925, 1.009]	1.171 [1.048, 1.309]
Birth interval < 12 months	1.113 [0.896, 1.383]	0.779 [0.460, 1.320]	0.957 [0.775, 1.182]	0.904 [0.481, 1.699]
Birth interval 12–23 months	1.146 [1.090, 1.205]	0.921 [0.819, 1.036]	1.097 [1.048, 1.148]	1.015 [0.867, 1.188]
Had diarrhea in last two weeks	1.121 [1.054, 1.192]	1.397 [1.251, 1.559]	1.180 [1.122, 1.242]	-
Maternal height < 160 cm	2.115 [1.819, 2.459]	1.105 [0.925, 1.319]	1.605 [1.446, 1.782]	1.215 [0.986, 1.498]
Maternal body mass index < 18.5 kg/m^2^	1.178 [1.137, 1.220]	1.608 [1.501, 1.723]	1.325 [1.286, 1.365]	1.109 [1.018, 1.209]
Age of mother at birth < 18 years	1.153 [1.086, 1.224]	1.122 [1.004, 1.254]	1.110 [1.057, 1.165]	1.164 [1.020, 1.329]
Age of mother at birth 18–19 years	1.040 [0.987, 1.096]	1.036 [0.932, 1.151]	1.020 [0.973, 1.069]	0.946 [0.829, 1.080]
Mother completed primary school	0.993 [0.953, 1.036]	1.041 [0.954, 1.136]	1.002 [0.966, 1.040]	0.902 [0.803, 1.012]
Mother completed secondary school	0.913 [0.864, 0.965]	0.922 [0.830, 1.024]	0.927 [0.885, 0.970]	0.835 [0.729, 0.956]
Mother completed tertiary level	0.704 [0.608, 0.815]	0.859 [0.704, 1.048]	0.745 [0.666, 0.833]	0.661 [0.520, 0.839]
Partner completed primary school	0.975 [0.936, 1.016]	1.045 [0.963, 1.134]	0.995 [0.960, 1.031]	0.941 [0.847, 1.047]
Partner completed secondary school	0.855 [0.809, 0.903]	1.062 [0.967, 1.166]	0.940 [0.899, 0.982]	0.888 [0.784, 1.006]
Partner completed tertiary level	0.688 [0.619, 0.764]	1.001 [0.857, 1.171]	0.818 [0.753, 0.888]	0.877 [0.725, 1.061]
Improved water source in household	0.950 [0.869, 1.039]	0.965 [0.812, 1.147]	0.946 [0.875, 1.022]	0.785 [0.623, 0.990]
Improved sanitation in household	0.958 [0.920, 0.998]	1.001 [0.930, 1.076]	0.967 [0.934, 1.000]	0.924 [0.843, 1.012]
Cooking with solid fuel	1.045 [0.949, 1.151]	0.948 [0.811, 1.107]	1.074 [0.990, 1.167]	1.053 [0.877, 1.264]
Urban residence	1.041 [0.990, 1.095]	0.934 [0.855, 1.020]	1.025 [0.984, 1.068]	1.102 [0.991, 1.225]
Wealth index quintile: poor	0.886 [0.846, 0.928]	0.978 [0.891, 1.073]	0.936 [0.901, 0.972]	0.946 [0.838, 1.069]
Wealth index quintile: middle	0.847 [0.804, 0.892]	0.886 [0.799, 0.982]	0.866 [0.828, 0.905]	0.921 [0.806, 1.052]
Wealth index quintile: rich	0.761 [0.715, 0.809]	0.811 [0.718, 0.916]	0.795 [0.754, 0.840]	0.853 [0.737, 0.987]
Wealth index quintile: richest	0.555 [0.506, 0.609]	0.789 [0.675, 0.923]	0.636 [0.589, 0.687]	0.872 [0.720, 1.056]
Survey fixed effect: 2007 wave	0.821 [0.777, 0.868]	1.220 [1.096, 1.357]	0.962 [0.916, 1.010]	-
Survey fixed effect: 2011 wave	0.835 [0.791, 0.882]	1.180 [1.060, 1.313]	0.938 [0.895, 0.982]	-
Survey fixed effect: 2014 wave	0.757 [0.712, 0.805]	1.076 [0.960, 1.207]	0.900 [0.854, 0.948]	1.118 [1.026, 1.218]
District fixed effects	Yes	Yes	Yes	Yes
N	23,187	23,188	23,187	11,870

Poisson regressions in which the reported coefficients are the estimated relative risk of the outcome and [95% confidence interval]. All regressions include 64 district fixed effects. Standard errors are robust and clustered at the Demographic and Health Survey (DHS) cluster level.

## Supplementary Materials

The following are available online at www.mdpi.com/1660-4601/15/1/22/s1, Table S1: Summary statistics for risk factors by child health outcomes: stunting, wasting, underweight, and small birth size, Tables S2–S5: Robustness checks for our results on child stunting, wasting, underweight, and small birth size, respectively. The first column in each table replicates the main results presented in Table 2 above. The second column adds interaction terms between PM_2.5_ exposure in utero and urban residence. The third column uses district level random effects rather than district level fixed effects. The fourth column uses district level random effects and controls for the mean PM_2.5_ level in the district in the child’s birth year.
