# Supplementary material for: Exposure to Ambient Fine Particulate Air Pollution in Utero as a Risk Factor for Child Stunting in Bangladesh
Source: Int J Environ Res Public Health. 2017 Dec 23;15(1):22. doi: 10.3390/ijerph15010022 (PMC5800122; doi:10.3390/ijerph15010022)
Supplement: Supplementary file 1 [file ijerph-15-00022-s001.pdf]

Supplementary Materials

Table S1. Summary statistics for risk factors by child health outcomes: stunting, wasting, underweight, and small birth size.

Variable, Outcome	Stunting	Wasting	Underweight	Small Birth Size
$PM_{2.5}$ exposure in utero				
19.6–40.2 µg/m³	5705 (24.60)	5706 (24.61)	5705 (24.60)	2233 (18.81)
40.3–45.8 µg/m³	5884 (25.38)	5884 (25.38)	5884 (25.38)	2863 (24.12)
45.9–51.9 µg/m³	5741 (24.76)	5741 (24.76)	5741 (24.76)	3074 (25.90)
52.0–72.9 µg/m³	5857 (25.26)	5857 (25.26)	5857 (25.26)	3700 (31.17)
Age of child (months)	30.22 (17.02)	30.22 (17.02)	30.22 (17.02)	-
Type of birth				
Singleton	22935 (98.91)	22936 (98.91)	22935 (98.91)	11700 (98.57)
Twin or triplet	252 (1.09)	252 (1.09)	252 (1.09)	170 (1.43)
Sex of child				
Male	11848 (51.10)	11849 (51.1)	11848 (51.1)	6119 (51.55)
Female	11339 (48.90)	11339 (48.9)	11339 (48.9)	5751 (48.45)
Birth order of child				
Second or higher	15783 (68.07)	15783 (68.07)	15783 (68.07)	7706 (64.92)
First	7404 (31.93)	7405 (31.93)	7404 (31.93)	4164 (35.08)
Short birth interval				
No	21125 (91.11)	21126 (91.11)	21125 (91.11)	10986 (92.55)
Yes, < 12 months	113 (0.49)	113 (0.49)	113 (0.49)	53 (0.45)
Yes, 12–23 months	1949 (8.41)	1949 (8.41)	1949 (8.41)	831 (7.00)
Had diarrhea in last two weeks				
No	21666 (93.44)	21667 (93.44)	21666 (93.44)	-
Yes	1521 (6.56)	1521 (6.56)	1521 (6.56)	-
Maternal height < 160 cm				
No	1000 (4.31)	1000 (4.31)	1000 (4.31)	557 (4.69)
Yes	22187 (95.69)	22188 (95.69)	22187 (95.69)	11313 (95.31)
Maternal body mass index < 18.5 kg/m²				
No	16370 (70.60)	16370 (70.60)	16370 (70.60)	8696 (73.26)
Yes	6817 (29.40)	6818 (29.40)	6817 (29.40)	3174 (26.74)
Teenage motherhood				
No	16279 (70.21)	16279 (70.20)	16279 (70.21)	8307 (69.98)
Yes, < 18 years	3606 (15.55)	3607 (15.56)	3606 (15.55)	1824 (15.37)
Yes, 18–19 years	3302 (14.24)	3302 (14.24)	3302 (14.24)	1739 (14.65)
Education level of mother				
None or incomplete primary	5718 (24.66)	5718 (24.66)	5718 (24.66)	2181 (18.37)
Completed primary	7052 (30.41)	7052 (30.41)	7052 (30.41)	3572 (30.09)
Completed secondary	8601 (37.09)	8602 (37.10)	8601 (37.09)	5109 (43.04)
Completed tertiary	1816 (7.83)	1816 (7.83)	1816 (7.83)	1008 (8.49)
Education level of mother's partner				
None or incomplete primary	7326 (31.60)	7326 (31.59)	7326 (31.60)	3266 (27.51)
Completed primary	6763 (29.17)	6764 (29.17)	6763 (29.17)	3562 (30.01)
Completed secondary	6226 (26.85)	6226 (26.85)	6226 (26.85)	3461 (29.16)
Completed tertiary	2872 (12.39)	2872 (12.39)	2872 (12.39)	1581 (13.32)

Variable, Outcome	Stunting	Wasting	Underweight	Small Birth Size
Improved water source in household				
No	702 (3.03)	702 (3.03)	702 (3.03)	260 (2.19)
Yes	22485 (96.97)	22486 (96.97)	22485 (96.97)	11610 (97.81)
Improved sanitation in household				
No	10289 (44.37)	10290 (44.38)	10289 (44.37)	5067 (42.69)
Yes	12898 (55.63)	12898 (55.62)	12898 (55.63)	6803 (57.31)
Type of cooking fuel in household				
Other	2706 (11.67)	2706 (11.67)	2706 (11.67)	1542 (12.99)
Solid fuel	20481 (88.33)	20482 (88.33)	20481 (88.33)	10328 (87.01)
Type of residence of household				
Rural	15854 (68.37)	15855 (68.38)	15854 (68.37)	8180 (68.91)
Urban	7333 (31.63)	7333 (31.62)	7333 (31.63)	3690 (31.09)
Wealth quintile of household				
Poorest	5219 (22.51)	5220 (22.51)	5219 (22.51)	2685 (22.62)
Poor	4532 (19.55)	4532 (19.54)	4532 (19.55)	2299 (19.37)
Middle	4362 (18.81)	4362 (18.81)	4362 (18.81)	2225 (18.74)
Rich	4368 (18.84)	4368 (18.84)	4368 (18.84)	2339 (19.71)
Richest	4706 (20.30)	4706 (20.29)	4706 (20.30)	2322 (19.56)
Demographic and Health Survey (DHS) Wave				
2004	5285 (22.79)	5286 (22.80)	5285 (22.79)	-
2007	4711 (20.32)	4711 (20.32)	4711 (20.32)	-
2011	6856 (29.57)	6856 (29.57)	6856 (29.57)	7668 (64.60)
2014	6335 (27.32)	6335 (27.32)	6335 (27.32)	4202 (35.40)
N	23,187	23,188	23,187	11,870

Values are mean (standard deviation) for continuous variables and count (percent) for categorical variables.

Table S2. The adjusted relative risk [95% confidence interval] of in utero $PM_{2.5}$ exposure on child stunting.

**Outcome: Stunting.**	**(1)**	**(2)**	**(3)**	**(4)**
$PM_{2.5}$ exposure in utero, 40.3–45.8 μg/m³	1.074 ** [1.014, 1.138]	1.069 ** [1.005, 1.138]	1.041 [0.970, 1.118]	1.061 [0.983, 1.145]
$PM_{2.5}$ exposure in utero, 45.9–51.9 μg/m³	1.150 *** [1.069, 1.237]	1.129 *** [1.045, 1.220]	1.070 [0.984, 1.164]	1.106 ** [1.003, 1.220]
$PM_{2.5}$ exposure in utero, 52.0–72.9 μg/m³	1.132 *** [1.031, 1.243]	1.114 ** [1.009, 1.231]	1.030 [0.933, 1.137]	1.085 [0.956, 1.232]
$PM_{2.5}$ exposure in utero, 40.3–45.8 μg/m³ * Urban	-	1.025 [0.916, 1.148]	-	-
$PM_{2.5}$ exposure in utero, 45.9–51.9 μg/m³ * Urban	-	1.089 [0.959, 1.236]	-	-
$PM_{2.5}$ exposure in utero, 52.0–72.9 μg/m³ * Urban	-	1.070 [0.939, 1.220]	-	-
Geometric average $PM_{2.5}$ level in district in birth year (μg/m³)	-	-	-	0.996 [0.990, 1.002]
Age of child (months)	1.013 *** [1.012, 1.014]	1.013 *** [1.012, 1.014]	1.013 *** [1.011, 1.014]	1.012 *** [1.011, 1.014]
Twin or triplet	1.616 *** [1.425, 1.832]	1.615 *** [1.424, 1.831]	1.610 *** [1.353, 1.915]	1.612 *** [1.355, 1.918]
Female	1.048 *** [1.014, 1.083]	1.048 *** [1.014, 1.083]	1.049 ** [1.005, 1.096]	1.049 ** [1.005, 1.096]
First child	0.968 [0.918, 1.020]	0.968 [0.918, 1.021]	0.968 [0.905, 1.035]	0.968 [0.905, 1.035]
Birth interval < 12 months	1.114 [0.897, 1.385]	1.113 [0.896, 1.383]	1.115 [0.841, 1.478]	1.114 [0.841, 1.478]
Birth interval 12–23 months	1.146 *** [1.090, 1.205]	1.146 *** [1.090, 1.205]	1.154 *** [1.073, 1.240]	1.152 *** [1.072, 1.239]
Had diarrhea in last two weeks	1.121 *** [1.054, 1.192]	1.121 *** [1.054, 1.192]	1.126 *** [1.037, 1.223]	1.126 *** [1.037, 1.223]
Maternal height < 160 cm	2.114 *** [1.818, 2.459]	2.115 *** [1.819, 2.459]	2.124 *** [1.807, 2.498]	2.124 *** [1.806, 2.497]
Maternal BMI < 18.5 kg/m²	1.178 *** [1.137, 1.220]	1.178 *** [1.137, 1.220]	1.185 *** [1.131, 1.242]	1.185 *** [1.131, 1.242]
Age of mother at birth < 18 years	1.153 *** [1.086, 1.224]	1.153 *** [1.086, 1.224]	1.146 *** [1.062, 1.237]	1.146 *** [1.062, 1.237]
Age of mother at birth 18–19 years	1.040 [0.986, 1.095]	1.040 [0.987, 1.096]	1.038 [0.967, 1.114]	1.039 [0.968, 1.115]
Mother completed primary school	0.994 [0.953, 1.036]	0.993 [0.953, 1.036]	0.990 [0.936, 1.048]	0.990 [0.936, 1.048]
Mother completed secondary school	0.914 *** [0.865, 0.965]	0.913 *** [0.864, 0.965]	0.904 *** [0.843, 0.969]	0.904 *** [0.843, 0.969]
Mother completed tertiary level	0.703 *** [0.607, 0.814]	0.704 *** [0.608, 0.815]	0.693 *** [0.593, 0.811]	0.693 *** [0.592, 0.811]
Partner completed primary school	0.975 [0.936, 1.016]	0.975 [0.936, 1.016]	0.978 [0.926, 1.033]	0.977 [0.926, 1.032]
Partner completed secondary school	0.855 *** [0.810, 0.903]	0.855 *** [0.809, 0.903]	0.854 *** [0.798, 0.914]	0.854 *** [0.798, 0.914]
Partner completed tertiary level	0.687 *** [0.618, 0.763]	0.688 *** [0.619, 0.764]	0.687 *** [0.610, 0.775]	0.687 *** [0.610, 0.775]
Improved water source in household	0.955 [0.873, 1.043]	0.950 [0.869, 1.039]	0.944 [0.837, 1.065]	0.945 [0.837, 1.066]
Improved sanitation in household	0.958 ** [0.920, 0.997]	0.958 ** [0.920, 0.998]	0.954 * [0.908, 1.003]	0.954 * [0.908, 1.003]
Cooking with solid fuel	1.043 [0.946, 1.150]	1.045 [0.949, 1.151]	1.009 [0.906, 1.123]	1.007 [0.904, 1.121]
Urban residence	0.999 [0.914, 1.092]	1.041 [0.990, 1.095]	1.043 [0.985, 1.104]	1.042 [0.984, 1.103]

**Outcome: Stunting.**	**(1)**	**(2)**	**(3)**	**(4)**
Wealth index quintile: poor	0.886 ***	0.886 ***	0.886 ***	0.885 ***
	[0.846, 0.928]	[0.846, 0.928]	[0.832, 0.942]	[0.832, 0.942]
Wealth index quintile: middle	0.847 ***	0.847 ***	0.848 ***	0.847 ***
	[0.804, 0.892]	[0.804, 0.892]	[0.792, 0.908]	[0.791, 0.907]
Wealth index quintile: rich	0.760 ***	0.761 ***	0.764 ***	0.764 ***
	[0.715, 0.809]	[0.715, 0.809]	[0.706, 0.827]	[0.706, 0.826]
Wealth index quintile: richest	0.555 ***	0.555 ***	0.558 ***	0.558 ***
	[0.506, 0.609]	[0.506, 0.609]	[0.501, 0.622]	[0.501, 0.622]
Survey fixed effect: 2007 wave	0.821 ***	0.821 ***	0.839 ***	0.844 ***
	[0.777, 0.868]	[0.777, 0.868]	[0.785, 0.898]	[0.789, 0.903]
Survey fixed effect: 2011 wave	0.835 ***	0.835 ***	0.854 ***	0.861 ***
	[0.791, 0.882]	[0.791, 0.882]	[0.802, 0.909]	[0.807, 0.917]
Survey fixed effect: 2014 wave	0.757 ***	0.757 ***	0.781 ***	0.790 ***
	[0.712, 0.805]	[0.712, 0.805]	[0.728, 0.837]	[0.735, 0.848]
Specification	District fixed effects	District fixed effects	District random effects	District random effects
N	23,187	23,187	23,187	23,187

Values are estimated coefficient [95% confidence interval]. * $p < 0.10$, ** $p < 0.05$, *** $p < 0.01$.

Table S3. The adjusted relative risk [95% confidence interval] of in utero $PM_{2.5}$ exposure on child wasting.

Outcome: Wasting	(1)	(2)	(3)	(4)
$PM_{2.5}$ exposure in utero, 40.3–45.8 μg/m³	1.109 * [0.990, 1.243]	1.123 * [0.987, 1.277]	1.004 [0.901, 1.119]	1.053 [0.933, 1.189]
$PM_{2.5}$ exposure in utero, 45.9–51.9 μg/m³	1.247 *** [1.082, 1.437]	1.188 ** [1.023, 1.379]	1.086 [0.969, 1.216]	1.185 ** [1.020, 1.377]
$PM_{2.5}$ exposure in utero, 52.0–72.9 μg/m³	1.272 *** [1.069, 1.512]	1.258 ** [1.051, 1.507]	1.049 [0.929, 1.186]	1.199 * [0.988, 1.456]
$PM_{2.5}$ exposure in utero, 40.3–45.8 μg/m³ * Urban	-	0.980 [0.797, 1.206]	-	-
$PM_{2.5}$ exposure in utero, 45.9–51.9 μg/m³ * Urban	-	1.256** [1.016, 1.554]	-	-
$PM_{2.5}$ exposure in utero, 52.0–72.9 μg/m³ * Urban	-	1.066 [0.848, 1.339]	-	-
Geometric average $PM_{2.5}$ level in district in birth year (μg/m³)	-	-	-	0.992 * [0.984, 1.001]
Age of child (months)	1.002 * [1.000, 1.004]	1.002 * [1.000, 1.004]	1.001 [0.999, 1.003]	1.001 [0.999, 1.003]
Twin or triplet	0.957 [0.659, 1.390]	0.958 [0.660, 1.392]	0.950 [0.670, 1.348]	0.953 [0.672, 1.351]
Female	1.017 [0.955, 1.083]	1.017 [0.955, 1.083]	1.019 [0.951, 1.091]	1.019 [0.951, 1.091]
First child	0.895 ** [0.812, 0.986]	0.895 ** [0.812, 0.986]	0.897 ** [0.808, 0.995]	0.896 ** [0.807, 0.994]
Birth interval < 12 months	0.779 [0.460, 1.320]	0.780 [0.460, 1.321]	0.779 [0.451, 1.344]	0.777 [0.450, 1.341]
Birth interval 12–23 months	0.921 [0.819, 1.036]	0.921 [0.819, 1.035]	0.918 [0.807, 1.044]	0.915 [0.804, 1.040]
Had diarrhea in last two weeks	1.397 *** [1.251, 1.559]	1.398 *** [1.253, 1.561]	1.406 *** [1.247, 1.584]	1.405 *** [1.247, 1.583]
Maternal height < 160 cm	1.105 [0.925, 1.319]	1.107 [0.927, 1.322]	1.106 [0.924, 1.323]	1.106 [0.924, 1.323]
Maternal body mass index< 18.5 kg/m²	1.608 *** [1.501, 1.723]	1.607 *** [1.500, 1.721]	1.610 *** [1.497, 1.732]	1.609 *** [1.496, 1.731]
Age of mother at birth < 18 years	1.122 ** [1.004, 1.254]	1.121 ** [1.002, 1.253]	1.129 * [1.000, 1.275]	1.132 ** [1.003, 1.279]
Age of mother at birth 18–19 years	1.036 [0.932, 1.151]	1.036 [0.932, 1.151]	1.038 [0.927, 1.162]	1.040 [0.929, 1.164]
Mother completed primary school	1.041 [0.954, 1.136]	1.042 [0.955, 1.137]	1.043 [0.949, 1.146]	1.045 [0.951, 1.148]
Mother completed secondary school	0.922 [0.830, 1.024]	0.923 [0.831, 1.025]	0.928 [0.830, 1.037]	0.930 [0.832, 1.039]
Mother completed tertiary level	0.859 [0.704, 1.048]	0.857 [0.702, 1.047]	0.861 [0.699, 1.061]	0.863 [0.700, 1.063]
Partner completed primary school	1.045 [0.963, 1.134]	1.045 [0.963, 1.134]	1.042 [0.952, 1.140]	1.041 [0.951, 1.139]
Partner completed secondary school	1.062 [0.967, 1.166]	1.062 [0.968, 1.166]	1.061 [0.954, 1.180]	1.060 [0.954, 1.179]
Partner completed tertiary level	1.001 [0.857, 1.171]	0.998 [0.854, 1.167]	1.000 [0.846, 1.182]	1.000 [0.846, 1.182]
Improved water source in household	0.965 [0.812, 1.147]	0.975 [0.820, 1.160]	0.920 [0.758, 1.116]	0.922 [0.760, 1.119]
Improved sanitation in household	1.001 [0.930, 1.076]	0.999 [0.928, 1.075]	1.004 [0.927, 1.087]	1.006 [0.929, 1.089]
Cooking with solid fuel	0.948 [0.811, 1.107]	0.940 [0.804, 1.099]	0.967 [0.829, 1.128]	0.963 [0.826, 1.124]
Urban residence	0.934 [0.855, 1.020]	0.869* [0.741, 1.020]	0.943 [0.861, 1.033]	0.941 [0.859, 1.030]

Outcome: Wasting	(1)	(2)	(3)	(4)
Wealth index quintile: poor	0.978	0.979	0.984	0.984
	[0.891, 1.073]	[0.892, 1.075]	[0.890, 1.090]	[0.889, 1.089]
Wealth index quintile: middle	0.886 **	0.887 **	0.908 *	0.906 *
	[0.799, 0.982]	[0.800, 0.983]	[0.813, 1.014]	[0.811, 1.012]
Wealth index quintile: rich	0.811 ***	0.810 ***	0.831 ***	0.830 ***
	[0.718, 0.916]	[0.717, 0.915]	[0.733, 0.942]	[0.732, 0.941]
Wealth index quintile: richest	0.789 ***	0.790 ***	0.813 **	0.813 **
	[0.675, 0.923]	[0.675, 0.924]	[0.693, 0.954]	[0.693, 0.953]
Survey fixed effect: 2007 wave	1.220 ***	1.220 ***	1.255 ***	1.257 ***
	[1.096, 1.357]	[1.097, 1.358]	[1.128, 1.397]	[1.130, 1.399]
Survey fixed effect: 2011 wave	1.180 ***	1.181 ***	1.223 ***	1.229 ***
	[1.060, 1.313]	[1.061, 1.314]	[1.104, 1.355]	[1.110, 1.361]
Survey fixed effect: 2014 wave	1.076	1.075	1.130 **	1.142 **
	[0.960, 1.207]	[0.959, 1.206]	[1.013, 1.261]	[1.023, 1.274]
Specification	District fixed effects	District fixed effects	District random effects	District random effects
N	23,188	23,188	23,188	23,188

Values are estimated coefficient [95% confidence interval]. * $p < 0.10$, ** $p < 0.05$, *** $p < 0.01$.

Table S4. The adjusted relative risk [95% confidence interval] of in utero $PM_{2.5}$ exposure on child underweight.

Outcome: Underweight	(1)	(2)	(3)	(4)
$PM_{2.5}$ exposure in utero, 40.3–45.8 μg/m³	1.118 *** [1.063, 1.176]	1.117 *** [1.057, 1.180]	1.050 [0.986, 1.118]	1.059 [0.988, 1.134]
$PM_{2.5}$ exposure in utero, 45.9–51.9 μg/m³	1.156 *** [1.087, 1.231]	1.138 *** [1.067, 1.215]	1.044 [0.972, 1.120]	1.059 [0.970, 1.156]
$PM_{2.5}$ exposure in utero, 52.0–72.9 μg/m³	1.127 *** [1.041, 1.220]	1.108 ** [1.019, 1.205]	0.995 [0.920, 1.076]	1.018 [0.910, 1.139]
$PM_{2.5}$ exposure in utero, 40.3–45.8 μg/m³ * Urban	-	1.015 [0.922, 1.116]	-	-
$PM_{2.5}$ exposure in utero, 45.9–51.9 μg/m³ * Urban	-	1.075 [0.970, 1.193]	-	-
$PM_{2.5}$ exposure in utero, 52.0–72.9 μg/m³ * Urban	-	1.075 [0.965, 1.197]	-	-
Geometric average $PM_{2.5}$ level in district in birth year (μg/m³)	-	-	-	0.999 [0.993, 1.004]
Age of child (months)	1.011 *** [1.010, 1.012]	1.011 *** [1.010, 1.012]	1.010 *** [1.009, 1.012]	1.010 *** [1.009, 1.012]
Twin or triplet	1.500 *** [1.339, 1.679]	1.500 *** [1.340, 1.679]	1.502 *** [1.278, 1.765]	1.503 *** [1.279, 1.766]
Female	1.092 *** [1.062, 1.123]	1.092 *** [1.062, 1.123]	1.094 *** [1.052, 1.137]	1.093 *** [1.052, 1.137]
First child	0.966 [0.925, 1.009]	0.966 [0.925, 1.009]	0.967 [0.911, 1.027]	0.967 [0.911, 1.027]
Birth interval < 12 months	0.957 [0.775, 1.182]	0.958 [0.776, 1.184]	0.952 [0.720, 1.258]	0.951 [0.720, 1.257]
Birth interval 12–23 months	1.097 *** [1.048, 1.148]	1.097 *** [1.048, 1.147]	1.103 *** [1.031, 1.179]	1.102 *** [1.030, 1.179]
Had diarrhea in last two weeks	1.180 *** [1.122, 1.242]	1.180 *** [1.122, 1.242]	1.186 *** [1.102, 1.276]	1.186 *** [1.102, 1.276]
Maternal height < 160 cm	1.605 *** [1.446, 1.782]	1.605 *** [1.446, 1.782]	1.615 *** [1.425, 1.830]	1.615 *** [1.425, 1.830]
Maternal body mass index < 18.5 kg/m²	1.325 *** [1.286, 1.365]	1.325 *** [1.286, 1.365]	1.335 *** [1.280, 1.392]	1.335 *** [1.280, 1.392]
Age of mother at birth < 18 years	1.110 *** [1.057, 1.165]	1.109 *** [1.056, 1.165]	1.104 *** [1.030, 1.182]	1.104 *** [1.031, 1.183]
Age of mother at birth 18–19 years	1.020 [0.973, 1.069]	1.020 [0.973, 1.068]	1.018 [0.955, 1.085]	1.018 [0.955, 1.085]
Mother completed primary school	1.002 [0.966, 1.040]	1.003 [0.966, 1.040]	0.997 [0.947, 1.050]	0.997 [0.947, 1.050]
Mother completed secondary school	0.927 *** [0.885, 0.970]	0.927 *** [0.885, 0.970]	0.917 *** [0.862, 0.977]	0.918 *** [0.862, 0.977]
Mother completed tertiary level	0.745 *** [0.666, 0.833]	0.744 *** [0.665, 0.832]	0.730 *** [0.640, 0.833]	0.730 *** [0.640, 0.833]
Partner completed primary school	0.995 [0.960, 1.031]	0.995 [0.960, 1.031]	0.996 [0.948, 1.047]	0.996 [0.948, 1.047]
Partner completed secondary school	0.940 *** [0.899, 0.982]	0.940 *** [0.899, 0.982]	0.939 ** [0.884, 0.997]	0.939 ** [0.884, 0.997]
Partner completed tertiary level	0.818 *** [0.753, 0.888]	0.817 *** [0.752, 0.887]	0.817 *** [0.738, 0.904]	0.817 *** [0.738, 0.904]
Improved water source in household	0.946 [0.875, 1.022]	0.950 [0.879, 1.027]	0.943 [0.846, 1.052]	0.944 [0.846, 1.053]
Improved sanitation in household	0.967 * [0.934, 1.000]	0.966 ** [0.934, 0.999]	0.964 [0.921, 1.008]	0.964 [0.921, 1.008]
Cooking with solid fuel	1.074 * [0.990, 1.167]	1.073 * [0.988, 1.166]	1.038 [0.944, 1.141]	1.037 [0.943, 1.140]

Outcome: Underweight	(1)	(2)	(3)	(4)
Urban residence	1.025	0.988	1.027	1.027
	[0.984, 1.068]	[0.920, 1.061]	[0.976, 1.081]	[0.976, 1.080]
Wealth index quintile: poor	0.936 ***	0.937 ***	0.939 **	0.939 **
	[0.901, 0.972]	[0.902, 0.973]	[0.887, 0.993]	[0.887, 0.993]
Wealth index quintile: middle	0.866 ***	0.866 ***	0.871 ***	0.871 ***
	[0.828, 0.905]	[0.828, 0.906]	[0.819, 0.927]	[0.818, 0.926]
Wealth index quintile: rich	0.795 ***	0.795 ***	0.805 ***	0.805 ***
	[0.754, 0.840]	[0.753, 0.839]	[0.750, 0.864]	[0.750, 0.864]
Wealth index quintile: richest	0.636 ***	0.636 ***	0.646 ***	0.646 ***
	[0.589, 0.687]	[0.589, 0.687]	[0.588, 0.710]	[0.588, 0.710]
Survey fixed effect: 2007 wave	0.962	0.962	0.988	0.989
	[0.916, 1.010]	[0.916, 1.010]	[0.930, 1.050]	[0.931, 1.051]
Survey fixed effect: 2011 wave	0.938 ***	0.937 ***	0.967	0.968
	[0.895, 0.982]	[0.895, 0.982]	[0.913, 1.023]	[0.914, 1.025]
Survey fixed effect: 2014 wave	0.900 ***	0.900 ***	0.938 **	0.941 *
	[0.854, 0.948]	[0.854, 0.948]	[0.882, 0.998]	[0.884, 1.001]
Specification	District fixed effects	District fixed effects	District random effects	District random effects
N	23,187	23,187	23,187	23,187

Values are estimated coefficient [95% confidence interval]. * $p < 0.10$, ** $p < 0.05$, *** $p < 0.01$.

Table S5. The adjusted relative risk [95% confidence interval] of in utero $PM_{2.5}$ exposure on small birth size.

Outcome: Small Birth Size	(1)	(2)	(3)	(4)
$PM_{2.5}$ exposure in utero, 40.3–45.8 μg/m³	1.040<br>[0.879, 1.230]	1.006<br>[0.829, 1.221]	0.987<br>[0.845, 1.152]	0.998<br>[0.847, 1.174]
$PM_{2.5}$ exposure in utero, 45.9–51.9 μg/m³	1.100<br>[0.906, 1.335]	1.040<br>[0.844, 1.282]	1.058<br>[0.894, 1.251]	1.081<br>[0.890, 1.314]
$PM_{2.5}$ exposure in utero, 52.0–72.9 μg/m³	1.209 *<br>[0.965, 1.516]	1.249 *<br>[0.987, 1.580]	1.169 *<br>[0.979, 1.397]	1.212<br>[0.954, 1.539]
$PM_{2.5}$ exposure in utero, 40.3–45.8 μg/m³ * Urban	-	1.087<br>[0.831, 1.423]	-	-
$PM_{2.5}$ exposure in utero, 45.9–51.9 μg/m³ * Urban	-	1.175<br>[0.907, 1.521]	-	-
$PM_{2.5}$ exposure in utero, 52.0–72.9 μg/m³ * Urban	-	0.799*<br>[0.613, 1.042]	-	-
Geometric average $PM_{2.5}$ level in district in birth year (μg/m³)	-	-	-	0.998<br>[0.987, 1.008]
Twin or triplet	2.205 ***<br>[1.720, 2.827]	2.215 ***<br>[1.725, 2.842]	2.230 ***<br>[1.736, 2.864]	2.233 ***<br>[1.739, 2.868]
Female	1.188 ***<br>[1.105, 1.278]	1.189 ***<br>[1.106, 1.279]	1.190 ***<br>[1.093, 1.295]	1.190 ***<br>[1.093, 1.295]
First child	1.171 ***<br>[1.048, 1.309]	1.172 ***<br>[1.049, 1.310]	1.175 **<br>[1.038, 1.330]	1.175 **<br>[1.038, 1.329]
Birth interval < 12 months	0.904<br>[0.481, 1.699]	0.897<br>[0.478, 1.684]	0.854<br>[0.425, 1.716]	0.852<br>[0.424, 1.712]
Birth interval 12–23 months	1.015<br>[0.867, 1.188]	1.016<br>[0.868, 1.190]	1.033<br>[0.872, 1.224]	1.032<br>[0.871, 1.223]
Maternal height < 160 cm	1.215 *<br>[0.986, 1.498]	1.221 *<br>[0.991, 1.505]	1.228 *<br>[0.978, 1.542]	1.228 *<br>[0.978, 1.542]
Maternal body mass index < 18.5 kg/m²	1.109 **<br>[1.018, 1.209]	1.109 **<br>[1.017, 1.209]	1.115 **<br>[1.014, 1.225]	1.114 **<br>[1.013, 1.225]
Age of mother at birth < 18 years	1.164 **<br>[1.020, 1.329]	1.165 **<br>[1.021, 1.330]	1.153 *<br>[0.997, 1.333]	1.153 *<br>[0.997, 1.333]
Age of mother at birth 18–19 years	0.946<br>[0.829, 1.080]	0.948<br>[0.831, 1.083]	0.938<br>[0.813, 1.082]	0.938<br>[0.814, 1.083]
Mother completed primary school	0.902 *<br>[0.803, 1.012]	0.899 *<br>[0.801, 1.009]	0.899 *<br>[0.795, 1.017]	0.899 *<br>[0.795, 1.017]
Mother completed secondary school	0.835 ***<br>[0.729, 0.956]	0.835 ***<br>[0.729, 0.956]	0.834 **<br>[0.726, 0.958]	0.834 **<br>[0.726, 0.958]
Mother completed tertiary level	0.661 ***<br>[0.520, 0.839]	0.663 ***<br>[0.521, 0.842]	0.651 ***<br>[0.505, 0.838]	0.651 ***<br>[0.505, 0.838]
Partner completed primary school	0.941<br>[0.847, 1.047]	0.942<br>[0.848, 1.048]	0.946<br>[0.844, 1.061]	0.946<br>[0.844, 1.061]
Partner completed secondary school	0.888 *<br>[0.784, 1.006]	0.892 *<br>[0.788, 1.010]	0.887 *<br>[0.776, 1.014]	0.887 *<br>[0.776, 1.014]
Partner completed tertiary level	0.877<br>[0.725, 1.061]	0.879<br>[0.727, 1.063]	0.872<br>[0.714, 1.066]	0.872<br>[0.714, 1.065]
Improved water source in household	0.785 **<br>[0.623, 0.990]	0.783 **<br>[0.621, 0.988]	0.793 *<br>[0.610, 1.031]	0.792 *<br>[0.610, 1.030]
Improved sanitation in household	0.924 *<br>[0.843, 1.012]	0.922 *<br>[0.841, 1.010]	0.928<br>[0.841, 1.024]	0.928<br>[0.841, 1.024]
Cooking with solid fuel	1.053<br>[0.877, 1.264]	1.032<br>[0.859, 1.241]	1.048<br>[0.872, 1.260]	1.048<br>[0.871, 1.259]
Urban residence	1.102 *<br>[0.991, 1.225]	1.102<br>[0.896, 1.355]	1.075<br>[0.961, 1.202]	1.074<br>[0.960, 1.202]
Wealth index quintile: poor	0.946<br>[0.838, 1.069]	0.947<br>[0.838, 1.070]	0.947<br>[0.833, 1.076]	0.947<br>[0.834, 1.077]
Wealth index quintile: middle	0.921<br>[0.806, 1.052]	0.925<br>[0.809, 1.057]	0.937<br>[0.815, 1.077]	0.937<br>[0.815, 1.077]

Outcome: Small Birth Size	(1)	(2)	(3)	(4)
Wealth index quintile: rich	0.853 **	0.849 **	0.871 *	0.871 *
	[0.737, 0.987]	[0.733, 0.983]	[0.745, 1.020]	[0.745, 1.019]
Wealth index quintile: richest	0.872	0.868	0.895	0.895
	[0.720, 1.056]	[0.717, 1.052]	[0.733, 1.093]	[0.733, 1.093]
Survey fixed effect: 2014 wave	1.118 **	1.115 **	1.118 **	1.123 **
	[1.026, 1.218]	[1.023, 1.214]	[1.021, 1.224]	[1.023, 1.233]
Specification	District fixed effects	District fixed effects	District random effects	District random effects
N	11,870	11,870	11,870	11,870

Values are estimated coefficient [95% confidence interval]. * $p < 0.10$, ** $p < 0.05$, *** $p < 0.01$.
